# Influence of Electricity upon Parturition

**Published:** 1870-04

**Authors:** 


M. de St. Germain presents to the same society the results of twelve experiments upon the influence of electricity upon parturition, as follows:

1st. In no case could uterine contractions be induced, unless they had already occurred spontaneously.

2nd. In each case in which the contractions, having already commenced, recurred at intervals of fifteen or twenty minutes, the application of the conductors to the lateral regions of the abdominal surface was followed in about ten minutes by a considerable acceleration in the uterine contractions.

3rd. Each contraction induced by electricity was of much longer duration and much more severe than the others.

4th. The dilatation of the neck appeared to proceed constantly with rapidity under the influence of galvanic excitation.

5th. In all the cases observed by us up to this time, the expulsion of the placenta has followed immediately that of the infant. It was either projected spontaneously outside of the vulva immediately after the expulsion of the fœtus, or was found in the vagina, and withdrawn without the least traction.

6th. Twice only was a slight bluish discoloration observed upon the infant, and in one of these the cyanosis could be attributed to the closely encircling cord. — *Gazette des Hopitaux.*

